# Ionone Derivatives from the Mycelium of *Phellinus linteus* and the Inhibitory Effect on Activated Rat Hepatic Stellate Cells

**DOI:** 10.3390/ijms17050681

**Published:** 2016-05-06

**Authors:** Shiow-Chyn Huang, Ping-Chung Kuo, Hsin-Yi Hung, Tai-Long Pan, Fu-An Chen, Tian-Shung Wu

**Affiliations:** 1 Department of Pharmacy, Chia-Nan University of Pharmacy and Science, Tainan 717, Taiwan; 2 School of Pharmacy, National Cheng Kung University Hospital, College of Medicine, National Cheng Kung University, Tainan 701, Taiwan; z10502016@email.ncku.edu.tw (P.-C.K.); z10308005@email.ncku.edu.tw (H.-Y.H.); 3 School of Traditional Chinese Medicine, Chang Gung University, Taoyuan 333, Taiwan; pan@mail.cgu.edu.tw; 4 Research Center for Industry of Human Ecology, Chang Gung University of Science and Technology, Taoyuan 333, Taiwan; 5 Liver Research Center, Chang Gung Memorial Hospital, Taoyuan 333, Taiwan; 6 Department of Pharmacy and Graduate Institute of Pharmaceutical Technology, Tajen University, Pingtung 907, Taiwan; fachen@tajen.edu.tw

**Keywords:** ionone, *Phellinus linteus*, fungus, phellinulin, hepatic fibrosis

## Abstract

Three new γ-ionylideneacetic acid derivatives, phellinulins A–C (**1**–**3**), were characterized from the mycelium extract of *Phellinus linteus*. The chemical structures were established based on the spectroscopic analysis. In addition, phellinulin A (**1**) was subjected to the examination of effects on activated rat hepatic stellate cells and exhibited significant inhibition of hepatic fibrosis.

## 1. Introduction

Hepatic fibrosis resulted from chronic liver injury induced by viral attack, autoimmune responses, drug-induced problems, cholestatic and metabolic diseases is a wound-healing response in which various cytokines and molecules would activate hepatic stellate cells (HSCs) and then undergo transformation from quiescent cells into fibrogenic cells [1,2,3,4,5,6,7,8]. Liver cirrhosis and further organ failure may occur with poor control of fibrosis [9]. The key intermediate, activated HSCs, can control the liberation of proinflammatory cytokines and tissue inhibitor of metallo-proteinases (TIMP) causing collagen deposition and further fibrosis [10]. Therefore, induction of activated HSCs apoptosis has been proposed as a potential anti-fibrotic strategy [11,12,13]. Emerging scientific evidences have suggested that traditional Chinese herbs are efficacious for treating chronic liver diseases due to their multi-ingredients, multi-mechanism of actions, and low adverse effects characteristics [14]. *Phellinus linteus* (Hymenochaetaceae) was usually called as “Sangwhang” in traditional Chinese medicines, and it has been utilized extensively as healthy foods or medicines in Asia to prevent various diseases, including cancer, ulcer, bacterial or viral infections and diabetes. In the literature, the fruiting bodies of *P. linteus* had been reported to possess bioactive compounds and display broadband bioactivities, such as cytotoxic, antioxidant, anti-inflammatory, anti-platelet aggregation, anti-dementia, anti-diabetic, and anti-viral activities [15,16,17,18,19,20]. Various aromatic hydrocarbons including caffeic acid, hydroxybenzaldehyde, hispidin, hispolon, and inotilone have also been characterized from the mycelium extracts and exhibited significant antioxidant and anti-inflammatory activities [21,22,23,24,25,26,27]. Recently, several articles reported the hepatoprotective and antihepatotoxic effects of the *Phellinus* genus [28,29,30,31,32]. In our preliminary study, the ethanol extract of cultured mycelium of *P. linteus* (PLE) exhibited protective effect against hepatic fibrosis in experimental animals. Therefore, in the present study we aimed to discover natural leads from *P. linteus* to inhibit hepatic fibrosis. The compositions of the constituents of the ethanolic mycelium extract were investigated. All isolated compounds as well as the crude extract were subjected to inhibition assay of activated HSCs to evaluate the potential for further development of treating liver fibrosis agents.

## 2. Results

### 2.1. Purification and Characterization

**Figure 1:**
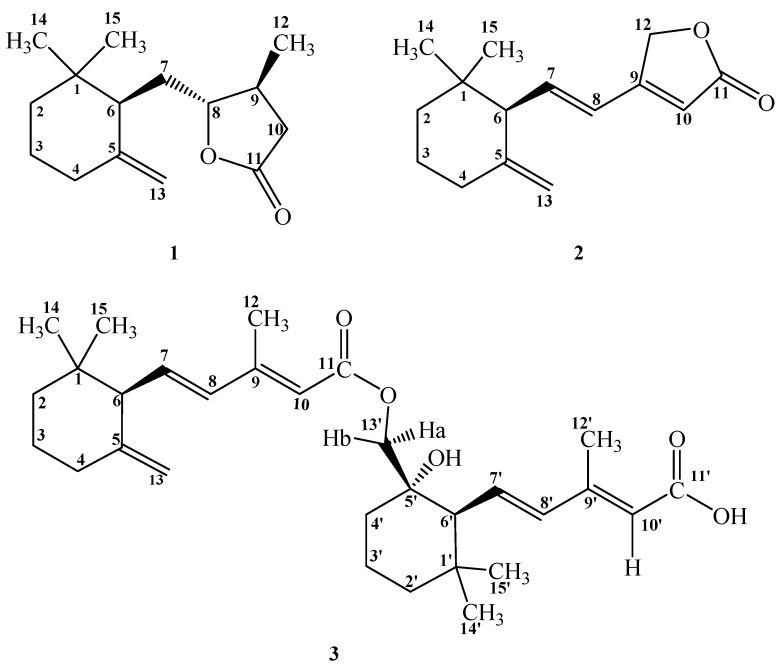
Chemical structures of phellinulins A–C (**1**–**3**).

The dried mycelium powder of *P. linteus* was refluxed with ethanol and the resulted extracts were partitioned with chloroform to afford CHCl_3_ and H_2_O soluble layers, respectively. The chloroform layer was purified by conventional chromatographic techniques to yield three new γ-ionylideneacetic acid derivatives (**1**–**3**) (Figure 1). Their structures were determined based on the 1D and 2D NMR, and mass spectrometric analytical results.

### 2.2. Structural Elucidation of Compounds ***1**–**3***

**Figure 2:**
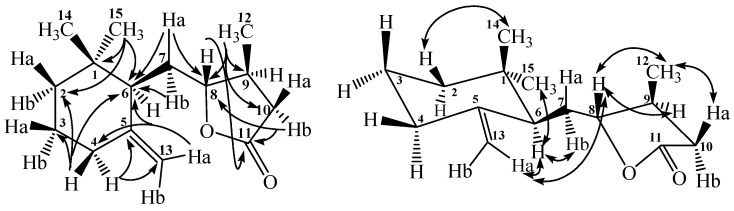
Significant HMBC (→) and NOESY (↔) correlations of compound **1**.

Compound **1** was afforded as colorless optically active powder, mp 60–63 °C and [α]_D_^25^ −111.6. The positive-mode HR-ESI-MS of **1** showed a pseudomolecular ion peak at *m*/*z* 259.1676 (calcd. for C_15_H_2__4_O_2_Na, 259.1674) constructing the molecular formula as C_15_H_2__4_O_2_. The IR absorption bands at 1782 and 1643 cm^−1^ indicated the presence of lactone carbonyl and carbon–carbon double bond functionalities, respectively. The ^1^H NMR spectrum of **1** displayed three methyls at δ 0.81 (3H, s, CH_3_-14), 0.93 (3H, s, CH_3_-15), and 1.10 (3H, d, *J* = 6.1 Hz, CH_3_-12). There were also five methylenes at δ 1.24–1.29 (1H, m, H-2a), 1.38–1.42 (1H, m, H-2b), 1.54 (2H, t, *J* = 6.0 Hz, H-3), 1.64 (1H, td, *J* = 12.0 and 3.2 Hz, H-7a), 1.75 (1H, td, *J* = 12.0 and 3.2 Hz, H-7b), 2.03 (2H, t, *J* = 6.0 Hz, H-4), 2.12–2.19 (1H, m, H-10a), 2.63 (1H, dd, *J* = 10.0 and 3.0 Hz, H-10b); and three methines at δ 2.12–2.19 (2H, m, H-6 & -9), 3.99 (1H, dd, *J* = 9.0 and 9.0 Hz, H-8), respectively. In the downfield region, there was also two olefinic protons at δ 4.61 (1H, br s, H-13a) and 4.83 (1H, br s, H-13b). The ^13^C NMR, DEPT-135 and HMQC spectral data displayed fifteen carbon signals including three methyl groups at δ 16.8, 25.7, 28.2, six methylene groups at δ 23.6, 31.1, 32.6, 36.4, 36.7, 110.0, three methines at δ 37.2, 49.8, 85.4, two quaternary carbons at δ 34.6, 148.0, and one carbonyl at δ 176.6, respectively. In the HMBC spectrum of **1** (Figure 2), there were ^2^*J*, ^3^*J*-correlations from CH_3_-14 and CH_3_-15 to C-1, C-2, and C-6; from CH_3_-12 to C-8, C-9, and C-10; from H-4 to C-2, C-3, C-5, C-6, and C-13; from H-7 to C-5, C-6, and C-8; from H-10 to C-8 and C-11; and from H-13 to C-4 and C-6, respectively. These spectral data established the structure of **1** as an ionone derivative. Moreover, HMBC correlation from H-8 to C-11 confirmed the presence of a γ-lactone ring linked through the oxygen atom between C-8 and C-11 was evidenced by IR absorption at 1782 cm^−1^.

In addition, there were NOESY correlations between H-6 and H-7, H-13, CH_3_-15; between H-8 and H-9, CH_3_-12, H-13; between H-10 and CH_3_-12; and between CH_3_-14 and H-2, respectively (Figure 2). These NOEs evidence the relative stereochemistry of **1** as shown. The successive two-dimensional spectral experiments accomplished the proton and carbon signal assignments of **1** and conclusively its chemical structure was determined as 5-((1,1-dimethyl-5-methylenecyclohexyl)-methyl)-4-methyl-dihydrofuran-2(3*H*)-one and named trivially as phellinulin A.

Compound **2** was obtained as optically active syrup and [α]_D_^25^ −70.4. In the HR-ESI-MS analytical data, **2** displayed a sodiated ion peak at *m*/*z* 255.1362 (calcd. for C_15_H_2__0_O_2_Na, 259.1361) and its molecular formula was determined as C_15_H_2__0_O_2_ with six degrees of unsaturation which is two more than that of **1**. The UV maximum at 260 nm and IR absorption bands at 1744 and 1647 cm^−1^ indicated the occurrence of an extended α, β-unsaturated carbonyl moiety. The ^1^H and ^13^C NMR spectral characteristics also suggested **2** to possess the ionone basic skeleton. The significant differences between **2** and **1** were only two methyl groups at δ 0.84 (3H, s, CH_3_-14) and 0.92 (3H, s, CH_3_-15), and three more olefinic protons at δ 5.86 (1H, s, H-10), 6.24 (1H, dd, *J* = 16.0 and 9.6 Hz, H-7), and 6.40 (1H, d, *J* = 16.0 Hz, H-8) could be found in **2**. In the HMBC spectrum of **2**, there were ^2^*J*, ^3^*J*-correlations from CH_3_-14 and CH_3_-15 to C-1, C-2, and C-6; from CH_2_-12 to C-9, C-10, and C-11; from H-4 to C-5 and C-13; from H-7 to C-8 and C-9; from H-8 to C-6, C-7, C-9, and C-12; from H-10 to C-11 and C-12; and from H-13 to C-4 and C-6 respectively. These spectral characteristics indicated that **2** is also an ionone derivative as **1**. The ^3^*J*-HMBC correlation from H-12 to C-11 constructed the formation of a γ-lactone ring between C-11 and C-12. NOE correlations of H-6/CH_3_-15 and H-7/CH_2_-12 in the NOESY spectrum determine the relative stereochemistry of **2**. The complete assignments of all the proton and carbon signals were furnished with the aid of other two-dimensional spectral analysis and therefore the chemical structure of **2** was constructed as (*E*)-4-(2-(1,1-dimethyl-5-methylenecyclohexyl)vinyl)furan-2(5*H*)-one and named trivially as phellinulin B.

Compound **3** was yielded as colorless optically active syrup with [α]_D_^25^ −84.0. The positive-mode HR-EI-MS of **3** showed a molecular ion peak at *m*/*z* 507.3086 corresponding to a molecular formula of C_30_H_44_O_5_. The mass spectrometric data implied the existence of an ionone dimer and it was supported by the ^1^H and ^13^C NMR in which showed spectral characteristics similar with the presence of two ionone units. The ^1^H NMR spectrum of **3** displayed six methyls, six aliphatic methylenes, and six olefinic protons; however, only one set of terminal olefinic methylene was found at δ 4.53 (1H, s, H-13a) and 4.76 (1H, s, H-13b). This indicated that the terminal olefinic methylene in one of the ionone units was replaced by other functional group and it was proved by the occurrence of one oxygenated methylene at δ 3.79 (1H, d, *J* = 11.0 Hz, H-13′a) and 3.97 (1H, d, *J* = 11.0 Hz, H-13′b). In the HMBC spectrum of **3**, there were ^2^*J*, ^3^*J*-correlations from CH_3_-14 and CH_3_-15 to C-1, C-2, and C-6; from CH_3_-12 to C-8, C-9, and C-10; from H-4 to C-13; from H-6 to C-1, C-5, C-7, and C-8; from H-7 to C-9; from H-8 to C-6, C-10, and C-12; from H-10 to C-8 and C-12; and from H-13′b to C-4′, C-5′ and C-11, respectively. In addition, there were ^2^*J*, ^3^*J*-correlations from CH_3_-14′ and CH_3_-15′ to C-1′, C-2′, and C-6′; from CH_3_-12′ to C-8′, C-9′, and C-10′; from H-6′ to C-1′, C-7′, and C-8′; from H-7′ to C-9′; from H-8′ to C-6′, C-10′, and C-12′; from H-10′ to C-8′ and C-12′, respectively. The ^3^*J*-HMBC correlation from H-13′a and H-13′b to C-11 (δ 167.1) determined the linkage of dimer to be through oxygen atom between C-11 and C-13′. The ^2^*J*-HMBC correlation from H-13′ to oxygenated quaternary carbon C-5′ (δ 73.0) also proved the presence of hydroxy group at C-5′. All the protons and carbons assignment and relative stereochemistry were performed with other 2D spectral analysis and the chemical structure of **3** was unambiguously constructed as shown (Figure 1) and named trivially as phellinulin C.

## 3. Discussion

**Figure 3:**
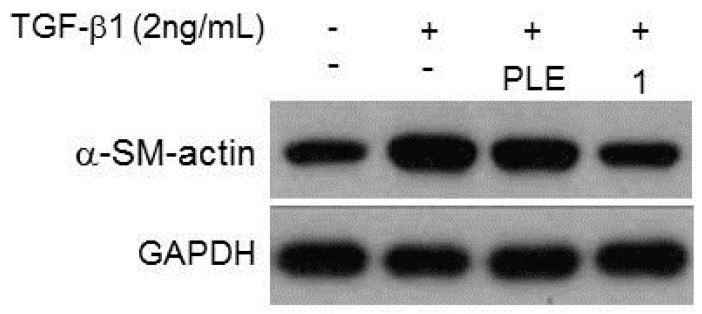
Western blot analysis of PLE and phellinulin A (**1**).

Plenty of evidences demonstrate that dimethylnitrosamine (DMN) and transforming growth factor-1 (TGF-1) can activate the quiescent hepatic stellate cells and transform them to proliferating myofibroblast-like cells to afford hepatic accumulation of extracellular matrix and result in liver fibrosis [33,34,35,36]. To evidence the inhibitory effect of PLE against *in vivo* hepatic fibrosis, the histological variations in rat liver tissues were examined and the results proved the protective effect (data not shown). Therefore, PLE and the purified compound phellinulin A (**1**) were further examined for their inhibitory activities on activated rat HSCs (Table 1) and the level of myofibroblast marker, α-smooth muscle actin (α-SM-actin; α-SMA) was evaluated [37]. At the tested concentration (40 μM), phellinulin A (**1**) exhibited the significant effects against the activated rat HSCs with the inhibition percentage of 67% (Figure 3). Thus the fruiting bodies of *P. linteus* could have potential for the inhibition of activated HSCs and develop as an anti-hepatic fibrosis drug in the future.

## 4. Materials and Methods

### 4.1. General

Melting points were recorded on a Yanagimoto MP-S3 apparatus (Kyoto, Japan) without corrections. Optical rotations were determined by the JASCO DIP-370 digital polarimeter (Tokyo, Japan). UV spectra were measured at room temperature with a Hitachi UV-3210 spectrophotometer (Tokyo, Japan), respectively. IR spectra were obtained with a Shimadzu FT-IR DR-8011 spectrophotometer (Kyoto, Japan). ^1^H and ^13^C NMR spectra were examined and recorded by a Bruker AV-500 NMR spectrometer (Billerica, MA, USA). Chemical shifts are expressed as δ values (ppm) using tetramethylsilane as an internal standard. The ESI-MS and HR-ESI-MS were taken on a Bruker Daltonics APEX II 30e spectrometer (positive-ion mode) (Billerica, MA, USA). The EI and HR-EI mass spectra were measured on a JEOL JMS-700 spectrometer (Tokyo, Japan). Column chromatography was conducted on 70–230 mesh and 230–400 mesh silica gels, and pTLC (preparative thin layer chromatography) was performed on Merck precoated Si gel 60 F254 plates (Darmstadt, Germany), and the spots on TLC was visualized by UV light.

### 4.2. Materials

The fermentation cultivated mycelium dried powder of *P. linteus* was provided and identified from Gene Ferm Biotechnology Co., Ltd. in Taiwan in August 2003. A voucher specimen (Wu 2003010001) has been deposited in the Herbarium of National Cheng Kung University, Tainan, Taiwan.

### 4.3. Extraction and Isolation

The dried mycelium powder of *P. linteus* (PL, 1.0 kg) was refluxed with ethanol (4 L × 5 × 4 h). The extract was then filtered with Whatman No. 1 filter paper, and the ethanol extracts were combined and concentrated at 40 °C under reduced pressure to obtain the ethanol extract (PLE, 500 g). PLE was further dissolved in water and partition with chloroform to give chloroform (PLEC, 220 g) and water extracts (280 g). The PLEC extract was purified by silica gel column chromatography eluted with *n*-hexane: ethyl acetate (4:1) to afford six fractions (Frs. 1–6). Fr. 3 was chromatographed on a silica gel column (chloroform: methanol = 29:1) to obtain **1** (21 mg) and **2** (3 mg), respectively. Fr. 5 was isolated by silica gel column chromatography with mixing eluent of 90% chloroform in acetone and further purified by pTLC to result in **3** (1 mg).

#### 4.3.1. Phellinulin A (**1**)

Colorless powder, mp 60–63 °C; [α]_D_^25^ −111.6; IR (neat) *v*_max_ 2932, 2870, 1782, 1643, 1215, 1150 cm^−1^; ^1^H NMR (CDCl_3_, 500 MHz) δ 0.81 (3H, s, CH_3_-14), 0.93 (3H, s, CH_3_-15), 1.10 (3H, d, *J* = 6.1 Hz, CH_3_-12), 1.24–1.29 (1H, m, H-2a), 1.38–1.42 (1H, m, H-2b), 1.54 (2H, t, *J* = 6.0 Hz, H-3), 1.64 (1H, td, *J* = 12.0, 3.2 Hz, H-7a), 1.75 (1H, td, *J* = 12.0, 3.2 Hz, H-7b), 2.03 (2H, t, *J* = 6.0 Hz, H-4), 2.12–2.19 (1H, m, H-6), 2.12–2.19 (2H, m, H-9 and H-10a), 2.63 (1H, dd, *J* = 10.0, 3.0 Hz, H-10b), 3.99 (1H, dd, *J* = 9.0, 9.0 Hz, H-8), 4.61 (1H, br s, H-13a), 4.83 (1H, br s, H-13b); ^13^C NMR (CDCl_3_, 125 MHz) δ 16.8 (C-12), 23.6 (C-3), 25.7 (C-14), 28.2 (C-15), 31.1 (C-7), 32.6 (C-4), 34.6 (C-1), 36.4 (C-2), 36.7 (C-10), 37.2 (C-9), 49.8 (C-6), 85.4 (C-8), 110.0 (C-13), 148.0 (C-5), 176.6 (C-11). ESI-MS *m*/*z* (*rel. int.*) 259 ([M + Na]^+^, 100); HR-ESI-MS *m*/*z* 259.1676 ([M + Na]^+^, calcd for C_15_H_24_O_2_Na, 259.1674).

#### 4.3.2. Phellinulin B (**2**)

Yellow syrup, [α]_D_^25^ −70.4; UV (MeOH) λ_max_ (log ε) 260 (4.71) nm; IR (neat) *v*_max_ 2936, 2866, 1744, 1647, 1451, 1150 cm^−1^; ^1^H NMR (CDCl_3_, 500 MHz) δ 0.84 (3H, s, CH_3_-14), 0.92 (3H, s, CH_3_-15), 1.33–1.39 (1H, m, H-2b), 1.47–1.51 (1H, m, H-2a), 1.55–1.63 (2H, m, H-3a and H-3b), 2.07 (1H, td, *J* = 14.0, 7.0 Hz, H-4a), 2.25 (1H, td, *J* = 14.0, 7.0 Hz, H-4b), 2.59 (1H, d, *J* = 9.6 Hz, H-6), 4.52 (1H, br s, H-13a), 4.80 (1H, br s, H-13b), 4.99 (2H, s, H-12), 5.86 (1H, s, H-10), 6.24 (1H, dd, *J* = 16.0, 9.6 Hz, H-7), 6.40 (1H, d, *J* = 16.0 Hz, H-8); ^13^C NMR (CDCl_3_, 125 MHz) δ 23.0 (C-3), 23.8 (C-14), 29.3 (C-15), 34.1 (C-4), 35.7 (C-1), 38.7 (C-2), 58.3 (C-6), 70.5 (C-12), 109.6 (C-13), 114.4 (C-10), 123.2 (C-8), 140.4 (C-7), 148.5 (C-5), 162.0 (C-9), 174.0 (C-11); ESI-MS *m*/*z* (*rel. int.*) 255 ([M + Na]^+^, 100); HR-ESI-MS *m*/*z* 255.1362 ([M + Na]^+^, calcd for C_15_H_20_O_2_Na, 255.1361).

#### 4.3.3. Phellinulin C (**3**)

Colorless syrup, [α]_D_^25^ −84.0; UV (MeOH) λ_max_ (log ε) 264 (4.57) nm; IR (neat) *v*_max_ 3499, 2943, 2870, 1694, 1609, 1242, 1150 cm^−1^; ^1^H NMR (CDCl_3_, 500 MHz) δ 0.82 (3H, s, CH_3_-15′), 0.83 (3H, s, CH_3_-15), 0.89 (3H, s, CH_3_-14), 1.06 (3H, s, CH_3_-14′), 1.19–1.21 (1H, m, H-2′a), 1.26–1.37 (2H, m, H-2a and H-4′a), 1.49–1.51 (2H, m, H-2′b and H-3′a), 1.56–1.60 (1H, m, H-2b), 1.71–1.78 (2H, m, H-3′b and H-4′b), 1.79 (1H, d, *J* = 10.0 Hz, H-6′), 1.97–2.06 (1H, m, H-4a), 2.17–2.26 (1H, m, H-4b), 2.30 (3H, s, CH_3_-12′), 2.31 (3H, s, CH_3_-12), 2.54 (1H, d, *J* = 9.4 Hz, H-6), 3.79 (1H, d, *J* = 11.0 Hz, H-13′a), 3.97 (1H, d, *J* = 11.0 Hz, H-13′b), 4.53 (1H, s, H-13a), 4.76 (1H, s, H-13b), 5.71 (1H, s, H-10), 5.74 (1H, s, H-10′), 6.08 (1H, d, *J* = 9.4 Hz, H-8′), 6.11 (1H, d, *J* = 9.4 Hz, H-8), 6.28 (1H, dd, *J* = 9.7, 7.8 Hz, H-7), 6.35 (1H, dd, *J* = 15.8, 10.0 Hz, H-7′); ^13^C NMR (CDCl_3_, 125 MHz) δ 14.2 (C-12′), 14.2 (C-12), 17.4 (C-3′), 22.3 (C-14′), 23.3 (C-3), 23.6 (C-15), 29.4 (C-14), 32.3 (C-15′), 34.4 (C-4), 34.4 (C-1′), 35.7 (C-4′), 35.7 (C-1), 39.0 (C-2), 41.2 (C-2′), 55.3 (C-6′), 58.1 (C-6), 71.4 (C-13′), 73.0 (C-5′), 108.9 (C-13), 117.1 (C-10′), 117.2 (C-10), 135.2 (C-8), 135.5 (C-7′), 136.9 (C-7 and -8′), 149.5 (C-5), 153.6 (C-9), 154.6 (C-9′), 167.1 (C-11), 170.4 (C-11′). EI-MS *m*/*z* (*rel. int.*) 507 ([M]^+^, 100); HR-EI-MS *m*/*z* 507.3086 ([M]^+^, calcd for C_30_H_44_O_5_, 507.3083).

### 4.4. Determination of the Inhibition Effect on Activated Rat Hepatic Stellate Cells

#### 4.4.1. Cell Culture and MTT Assay

The immortalized rat myofibroblast cell line HSC-T6 was a gift kindly provided by Dr. Scott L. Friedman (Mount Sinai School of Medicine, New York, NY, USA). The HSC-T6 cells were cultured in Waymouth medium with 10% fetal bovine serum (FBS) at 37 °C in a humidified atmosphere of 5% CO_2_. HepG2 cells were cultured with Dulbecco’s Modified Eagle’s medium (DMEM) containing 10% FBS for 24 h. Cell viability was examined by MTT assay. Totally 1 × 10^5^ cells were seeded in 24-well plates for 24 hours (h) and made quiescent by cultivation in medium containing 0.2% FBS overnight. After treating with 40 μM of phellinulin A (**1**) for 72 h, tetrazolium salt mixed isopropanol solution was added to the wells and the resulting mixture was more incubated at 37 °C for 4 h [34]. The optical density of the dissolved solution was recorded at 570 nm by the spectrophotometer, and all the experiments were performed at least in triplicate.

#### 4.4.2. Western Blot Analysis

Proteins were isolated on 10% denatured gels and transferred to PVDF membranes and then incubated for 1 h with blocking solution of 5% nonfat milk-TBST solution. The membranes were further immersed in the same solution containing an antibody against glyceraldehyde 3-phosphate dehydrogenase (GAPDH) and α-SMA (Santa Cruz, Dallas, TX, USA) overnight. After washing with the mixture of tris-buffered saline and Tween^®^ 20 (TBST) (Cell Signaling Technology, Danvers, MA, USA) four times, the membranes were cultured with the 5% nonfat milk-TBST solution containing peroxidase-labeled anti-rabbit IgG (Santa Cruz, Dallas, TX, USA) for 2 h. After washing in TBST five times, enhanced chemiluminescence substrate (ECL) (PerkinElmer™, Waltham, MA, USA) was used for protein detection. The intensity of each band was quantified using GeneTools Image Software (Syngene, Cambridge, UK), as GAPDH was used as the internal control. The Western blot experiments were conducted in triplicate.

#### 4.4.3. Statistical Analysis

The experimental data were expressed as the mean ± standard deviation (SD) of three parallel measurements (SigmaPlot 8.0; SPSS Inc., Chicago, IL, USA).

## Figures and Tables

**Table 1:** Inhibitory effects of *P. linteus* (PLE) and phellinulin A (**1**) on activated rat hepatic stellate cells (HSCs).

Sample	Cell Viability (Fold of Base)	Inhibition Percentage (%)
Control	0.53 ± 0.01	–
TGF	1.65 ± 0.03	–
TGF + PLE ^a^	1.19 ± 0.02	28%
TGF + **1** ^b^	0.54 ± 0.01	67%

The data were presented as mean ± S.D. ^a^ Test concentration: 40 μg/mL; ^b^ Test concentration: 40 μM; –: Not determined.
